# Maturation strategies and limitations of induced pluripotent stem cell-derived cardiomyocytes

**DOI:** 10.1042/BSR20200833

**Published:** 2021-06-16

**Authors:** Peng Wu, Gang Deng, Xiyalatu Sai, Huiming Guo, Huanlei Huang, Ping Zhu

**Affiliations:** 1 Department of Cardiac Surgery, Guangdong Cardiovascular Institute, Guangdong Provincial People’s Hospital, Guangdong Academy of Medical Sciences, Guangzhou, Guangdong, China; 2 The Second School of Clinical Medicine, Southern Medical University, Guangzhou, Guangdong, China

**Keywords:** cardiomyocytes, induced pluripotent stem cells, limitations, maturation, strategies

## Abstract

Induced pluripotent stem cells (iPSCs) have the ability to differentiate into cardiomyocytes (CMs). They are not only widely used in cardiac pharmacology screening, human heart disease modeling, and cell transplantation-based treatments, but also the most promising source of CMs for experimental and clinical applications. However, their use is largely restricted by the immature phenotype of structure and function, which is similar to embryonic or fetal CMs and has certain differences from adult CMs. In order to overcome this critical issue, many studies have explored and revealed new strategies to induce the maturity of iPSC-CMs. Therefore, this article aims to review recent induction methods of mature iPSC-CMs, related mechanisms, and limitations.

Cardiovascular disease (CVD) is the leading cause of morbidity and mortality worldwide. Among them, acute myocardial infarction (AMI) is the most common type of CVD [1]. Due to the limited ability of the heart to metabolize hypoxia, ischemia, and hypoxia of cardiac cells (cardiomyocytes, CMs) cause a series of adverse events, including apoptosis, necrosis, various inflammatory reactions and remodeling, scar formation, and eventually develop into end-stage heart disease [2]. Although drugs, percutaneous coronary intervention (PCI) and surgery can significantly slow the progression of the disease and improve the patient’s prognosis, none of the current treatments can achieve the regeneration of damaged tissue or myocardium [3]. Heart transplantation is the only effective final treatment for ischemic heart failure caused by MI. However, due to scarcity of donors, difficult surgery, high risk, and related complications such as postoperative immune rejection, it has greatly limited its application and promotion [4]. Stem cells (SCs) are a group of cells with self-renewal ability and multiple differentiation ability, which has become a prevailing method for treating MI [5]. In addition, SCs also have powerful paracrine functions, which can reduce cell death, increase angiogenesis, regulate inflammatory responses, and stimulate CMs proliferation [6].

Bone marrow mesenchymal stem cells (BM-MSCs), adipose-derived stem cells (ADSCs), and cardiac stem cells (CSCs) have been used in clinical trials and have been proven to be safe and effective for treating MI patients. Recent studies have shown that the therapeutic effect of these cells mainly depends on paracrine function, but the ability to differentiate into CMs is weak, and the retention time after transplantation is short [7]. Induced pluripotent stem cells (iPSCs) were introduced by Yamanaka et al. [8] to induce somatic cell reprogramming through Oct3/4, Sox2, c-Myc, and Klf4 factors, and have similar multipotent potentials as embryonic stem cells (ESCs). It turns out that iPSCs transplantation can effectively reduce the infarct size and improve cardiac function after MI [9,10]. Studies have shown that in rat models of MI, iPSCs injected into the myocardium can differentiate into CMs, smooth muscle cells, and ECs [11]. Among them, differentiated CMs can be integrated into the tissue structure of the rat myocardium [12]. Even so, because the cell population contains a certain percentage of undifferentiated cells, the risk of teratoma formation increases with the number of transplanted cells. It has been reported that ESCs [13] and iPSCs [14] have tumorigenic effects after undifferentiated transplantation. In contrast, no tumorigenicity has been reported after iPSC-CMs transplantation, and it also has a cardiac function improvement in rodent, pig, and non-human primate MI models [15–18]. In addition, a research compared the efficacy of iPSC-CMs with somatic stem cells (skeletal myoblasts, MSCs) after MI treatment in large animals had found that iPSC-CMs significantly increase angiogenesis density in infarcted areas, reduce CMs apoptosis, reduce the wall stress, increase the metabolic efficiency of the heart, and better improve cardiac function after infarction [19]. The above studies suggest that iPSC-CMs are superior to other somatic sources in improving cardiac metabolism and function after MI, and have the greater clinical benefit for severely damaged myocardium.

Nevertheless, the treatment strategies based on iPSC-CMs are inadequate. Since immature iPSC-CMs exhibit the electrophysiological and spontaneous beating characteristics of fetal-like CMs, they may not match the electrophysiological characteristics of the host myocardium after transplantation, resulting in arrhythmia [20]. The problems are that they still have the largest depolarized diastolic potentials [21], mitochondrial dysfunction [22], the expression of cardiac maturation marker cTnI is down-regulated and other immature manifestations [23]. *In vivo*, although the transplanted CMs are mature in a sense [24], the expression of myocardial-specific proteins is weaker than that of the host myocardium and shows significantly different tissue structure. In addition, the expression of gap junction (GJ) proteins, such as connexin 43 (Cx43), is relatively low in iPSC-CMs, and the electrical conduction is uneven as the heart rhythm increases [16,25], this electrical heterogeneity is also one of the main causes of arrhythmia after transplantation [16,24].

Therefore, in this literature, we will compare the differences between iPSC-CMs and adult CMs (Table 1), review the strategies for inducing iPSC-CMs to mature, and the shortcomings.

**Table 1:** Comparison of iPSC-CMs and adult CMs

	Parameters	iPSC-CMs	Adult CMs
Morphology	Cell shape	Circular	Rod-shaped
	Size	Small	Large
	Nuclei	Mononuclear	25–30% Binuclear
Ultrastructure	Sarcomere	1.6 μm	2.2 μm
	T-tubules	Absent	Present
	Mitochondria	Small, slender and long, close to nucleus and at periphery, lack mitochondrial cristae	Ovular shape, 20–40% of cell volume, arranged between myofibrillar and submuscular membranes
Metabolism		Glucose/glycolysis	Fatty acid/β-oxidation
Electrophysiology	RMP	−50 to −60 mV	−90 mV
	Upstroke velocity	15–50 V/s	230–400 V/s
	GJ	Around all sides of the cell membrane	Intercalated discs
	Conduction velocity	10–20 cm/s	60 cm/s
Myofibrillar isoform	Titin	N2BA	N2B
	MHC	β≈α	β>>α
Up-regulated genes in adult CMs	Sarcomere		MYL2, TNNI3, ACTN2, MYH7, MYL3, TNNC1, TNNT2, MYH11, SORBS1
	Ion transporters and their regulatory proteins at sarcolemma		KCNA4, KCNA5, KCNAB1, KCNAB2, KCND2, KCND3, KCNE4, KCNG1, KCNH2, KCNH7, KCNIP2, KCNJ2, KCNJ3, KCNJ5, KCNJ8, KCNK1, KCNQ1, KCNV1, SCN1A, SCN1B, SCN2B, SCN3A, SCN4B, SCN5A, HCN1, HCN4, CACNA1C, CACNA1D, CACNA1H, CACNA1G, CACNA2D1, CACNB2, SLC8A1, TRPC3, TRPC4, TRPC6, CFTR
	Ion transporters and their regulatory proteins at SR		ATP2A2, PLN, CASQ2, RYR2, RYR3, TRDN, ITPR1, ITPR3, ASPH, S100A1, HRC

## Differences between immature iPSC-CMs and adult CMs

### Morphology

Although strategies for improving iPSC-CMs have been continuously explored before this, there are still differences between them and adult CMs (Table 1). As early as 2009, researchers have differentiated iPSCs into beating embryonic body (EB), and expressed myocardial-specific genes such as NKX2.5 and myofibrin [21]. Nonetheless, iPSC-CMs still exhibit immature morphology and are similar to fetal CMs. They are round, smaller than adult CMs, and mostly mononuclear. In contrast, adult CMs are rod-shaped, anisotropic, and have a high aspect ratio, with 25–30% being binucleated [26,27]. The sarcomere is a contractile unit of CMs, the assembly and turnover of the cardiac sarcomere is essential for cardiac function because it contains the essential contractile components of actin and myosin, which are necessary for cardiac function [28]. The iPSC-CMs lack clear T-tubes, show a disordered sarcomere stripes, have a short length (1.6 μm), contain partially irregularly arranged myofibrils, and most cells only have immature high-density Z-band and I-band [26,29,30]. After staining cardiac troponin T (cTnT) and cTnI in adult CMs, the highly organized and slender sarcomere structure (2.2 μm), α-actin, and β-myosin heavy chain (β-MHC) can be seen, in which β-MHC expression is much higher than α-MHC [26,27]. Using iPSC-CMs to characterize the basic mechanism of cardiac sarcomere formation, it was found that β-cardiac myosin–titin–protocostamere forms an indispensable mechanical connection, and the force transmitted by this mechanism directly guides α-actinin-2 centripetal fiber assembly and sarcomere formation. Titin transmits diastolic traction from β-cardiac myosin instead of α-cardiac myosin or non-muscle myosin II to protocostameres during sarcomere formation [31]. Therefore, understanding the morphological differences and formation mechanisms between iPSC-CMs and adult CMs is essential for the function of CMs, and also provides a reference for the subsequent induction of CM maturation. After long-term cultivation of iPSC-CMs, the arrangement, density, and morphology of myofibrils have been significantly improved, showing the Z-disk, A-band, I-band, and H-regions of adult-like CMs [32,33].

Correspondingly, adult CMs express important structural genes at high levels, such as myosin heavy chain 7 (MYH7), cardiac titin (N2B), cTnI and sarcoplasmic reticulum ATPase (SERCA2). In contrast, iPSC-CMs showed high levels of MYH6, not MYH7, and expressed the N2A subtype of cardiac adiponectin rather than N2B [26]. In addition, there are still most genes that are highly expressed in adult CMs [34,35]. The two main isoforms of myosin light chain (MLC) are MLC2a and MLC2v, which are encoded by the genes *MYL7* and *MYL2*. Among them, MLC2a positive refers to atrial-like CMs, MLC2v positive refers to ventricular-like CMs, and MLC2v positive/MLC2a negative refers to mature ventricular-like CMs [36–38]. In iPSC-CMs, both isoforms are expressed, which may be due to the presence of a mixture of atrial and ventricular myocytes in the cell population [30,39].

### Mitochondria and metabolism

CMs continue to multiply in the first few months after human birth. The increase in contractile components requires an increase in ATP production. As a result, mitochondrial biogenesis is induced, and energy metabolism is converted from consumption of glucose and lactic acid into β-oxidation of fatty acids produced by more energy [40]. In the early stages of postnatal development of the heart, due to this increased biogenesis, the relative mitochondrial mass of CMs has doubled [41]. A large number of mitochondria are uniformly distributed in adult CMs, accounting for 20–40% of the cell volume [26]. The mitochondria of adult CMs are oval and have lamellar cristae, arranged between myofibrils and submuscular membranes, to effectively provide ATP for contraction and ion pumping [42,43]. However, mitochondria in iPSC-CMs are located in the perinuclear space, are small, slender, and lack mitochondrial cristae [42].

Mature mitochondria are the powerhouses of cells, and an extensive surface area provided by dense mitochondrial cristae promotes effective cellular respiration [44]. Recent studies have reported that converting metabolism of CMs into an oxidative pathway can promote cell maturation, thereby affecting sarcomere length and Ca^2+^ transient dynamics [45–47]. As mentioned earlier, iPSC-CMs exhibit the characteristics of fetal-like CMs [20], cell proliferation is active, and even in the presence of sufficient oxygen, the metabolism of energy substrates in proliferating cells has a high rate of glycolysis and lactic acid production. This metabolic phenotype is suitable for the biosynthesis of cellular lipids, amino acids, nucleotides, and other macromolecules [48]. So in immature iPSC-CMs, metabolism depends on glycolysis, but because adult CMs continue to require a lot of energy to maintain normal heart function, fatty acid β-oxidation is used as an effective way for ATP synthesis [49,50].

### Ca^2+^ handling

In adult CMs, Ca^2+^ plays a major role in contraction, signaling, metabolism, and transcriptional regulation. In the excitation–contraction coupling, depolarization propagates along the sarcolemma and its intima (T-tube), leading to the opening of L-type calcium channels (LTCCs), causing extracellular Ca^2+^ to flow into the cell. As a result, calcium-induced Ca^2+^ is released from sarcoplasmic reticulum (SR) through ryanodine receptors (RYRs). Once released from the SR, the binding of calcium to troponin C initiates myofilament sliding and muscle contraction. During diastole, calcium is recaptured to SR through SERCA2a and pumped out of the cell through Na^+^–Ca^2+^ exchanger, with little mitochondrial absorption. This effective calcium treatment system is mediated by the specific spatial organization of the calcium treatment structure. It tightly combines LTCCs with RYRs on the SR, forming an effective excitation–contraction coupling hub in the length and width of the cell [51]. In contrast, in iPSC-CMs, there is no T-tube, and SR is not developed, and the expression of SERCA and other key proteins is low. As a result, iPSC-CMs relies on LTCC to increase Ca^2+^, while excitation–contraction coupling is very slow [52,53].

### Electrophysiology

The lipid bilayer membrane of adult CMs is embedded with an integrin called an ion channel, which can promote the passive diffusion of ions on the hydrophobic cell membrane. There are a large number of negatively charged molecules (mainly proteins), and energy-consuming membrane pump activities (mainly Na^+^/K^+^-ATPase and Ca^2+^-ATPase). As a result, the distribution of charged ions is uneven and a transmembrane electrochemical gradient is generated, thereby generating a membrane potential (MP). In the resting state, because a certain type of K^+^ channel (Kir2.1) is always open, causing K^+^ outflow, the resting MP (RMP) of CMs tends to be equal to the K^+^ equilibrium potential of −90 mV [54]. Action potential (AP) is the result of the opening and closing of multiple ion channels on the CMs membrane. In phase 0, the rapid sodium influx (*I*Na) depolarizes the cell. Subsequently, during the initial phase of repolarization (Phase 1), the transient outward potassium current (*I*To1) was activated to cause K^+^ outflow. Ca^2+^ (*I*Ca, L) caused the AP to enter the platform phase (Phase 2). The activation of the fast and slow-delayed rectified K^+^ channels (*I*Kr and *I*Ks) repolarizes the membrane (Phase 3). Inward rectification of the K^+^ channel (*I*K1) helps to maintain the RMP in Phase 4 [55,56]. Relatively, iPSC-CMs show a negative decrease in maximum diastolic potential (MDP) in electrophysiology, the AP rises slowly, and the initial repolarization is not obvious [21,57,58]. In addition, the significant differences between iPSC-CMs (atrial, ventricular-like cells) and adult CMs include spontaneous beating [57] due to the reduced density of *I*K1 and the expression of the pacing current (*I*f) of iPSC-CMs [59,60]. Recently, some studies have revealed that increasing the expression of Kir2.1 in iPSC-CMs can improve AP and Ca^2+^ handling at the same time [60]. Similarly, the integration of biological and informatics technologies for *I*K1 current signal conversion can induce more physiological and stable MDP, ventricular-like AP, and increase the upstroke velocity of Phase 0, closer to the electrophysiological characteristics of adult CMs [61,62].

### Conduction velocity

The electrical signal conduction velocity mainly depends on the Na^+^ channel. Because iPSC-CMs lack this ion channel, the electrical signal conduction velocity is slower than adult CMs (10–20 vs 60 cm/s) [26,63,64]. In addition to ion channels, the composition and distribution of GJs also have important effects on conduction velocity. Cx43 is involved in the formation of ventricular GJs. In adult CMs, Cx43 and Na^+^ channels are clustered at the intercalated disc, and the side connections of the cells are rare [65]. In contrast, in iPSC-CMs, GJ is distributed around all sides of the cell membrane, and is rarely located at the intercalated disc [66]. In addition, the conduction velocity is also positively correlated with cell size, which may be one of the reasons for the slow conduction velocity of iPSC-CMs [67]. Since the iPSC differentiated cell population contains a small number of other cell types, it also slows down the conduction velocity [68].

## Strategies for inducing iPSC-CMs to mature

**Figure 1:**
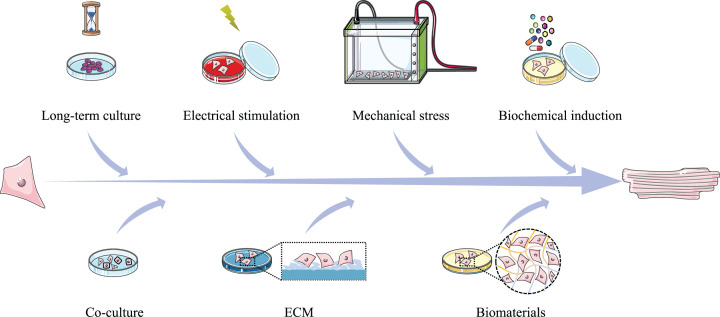
Summary of strategies to promote the maturity of iPSC-CMs

With the continuous research, many methods have been found to promote the production of mature iPSC-CMs. Here, we summarize the measures recently established (Figure 1 and Table 2).

**Table 2:** Summary of different maturation approaches for iPSC-CMs

Stimulation type	Specific approach	Effects of stimulation	References
Time	Long-term culture	Increased cell size, anisotropy, and myofibril density	[32,33]
		More mature Ca^2+^ handling, MDP and AP	
		Appearance of Z, A, H, I, and M bands	[33]
Physical	Electrical	The expression of specific myocardial structural proteins and the level of functional maturation-related genes are up-regulated. iPSC-CMs showed mature sarcomere structures, and intracellular calcium levels increased significantly	[71]
		Increaed cTnT expression, showed sarcomere-like structures	[72]
		Improving the integration of grafts with host CMs by promoting the expression of GJ proteins. Promote the release of Ca^2+^, related gene expression and regulate the contractile function	[73,75]
		More mature cell morphology and muscle fiber network	[75]
		Improving mitochondrial alignment	[76]
	Mechanical stress	Increased CMs and matrix fiber alignment and enhances myofibrillogenesis and sarcomeric banding	[77]
	Increased afterload	Improving CMs morphology and sarcomere length, improved Ca^2+^ handling, increased expression of several key markers of maturation	[78]
	Passive Stretch	Improving sarcomere alignment, Ca^2+^ handling, increased expression of Ca^2+^ and K^+^ channel-related genes	[79]
	Uniaxial strain	More uniform sarcomere orientation	[80]
	Combined electromechanical	Enhanced membrane N-Cad signal and stress-fiber formation	[81]
Biochemical	T3	Increased cell size, sarcomere length, contractility and SERCA2a expression, improving Ca^2+^ handling and mitochondrial function	[22]
	T3+Dex	Promote T-tube formation, Ca^2+^ release, and enhanced functional coupling between LTCCs and RYR2	[85]
	Fatty acid	Increased the number of mitochondria, oxidative metabolism and myofibril density, more regular Z-line arrangement, improved Ca^2+^ handling, enhanced CMs contractility, higher AP time course, APD50/APD90 ratio and upstroke velocity	[85,87,88]
		Inhibition of HIF-1α and its target LDHA	[45]
	TID	More mature energy metabolism and electrophysiology	[84,90]
	TID+HIF-1α inhibitor+PPARα agonist		
	Torin1	Enhancing contractility, metabolic maturation and expression of mature ion channels, increased peak rise time and downstroke velocity, which is related to the cell quiescence induced by p53	[96]
	β-AR	More mature sarcomere alignment and SR	[98]
	NRG-1β	More mature AP	[99]
3D tissue engineering	Co-culture	More mature SR and contractility	[100]
		Improved cell arrangement, sarcomere structure, and contractility	[101]
		More mature ultrastructure, electromechanical coupling, and Ca^2+^ handling	[103]
		The sarcomere structure and T-tube structure have been improved, and energy metabolism and electrophysiology have become more mature	[105]
		Improving the morphology and electrical integration through soluble factors and exosomes	[106]
	ECM	Promote electrophysiological maturation and functional GJs between cells	[113]
		Induced maturation of CMs structure, expression of Ca^2+^ handling and ion channel related genes	[114,115]
	dcECM	Improve the structure and contractility of CMs and increase the expression of related proteins	[117]
		Improve cell contraction and metabolism	[118]
	Biomaterials	Formation of intercellular networks, maturation of cell structure, increased contractility and expression of related proteins	[120]
		Increase sarcomere length and cell contraction, enhance Ca^2+^ handling capacity	[121,124]
		More mature structure and electrophysiology, promote the transition from E-Cad to N-Cad	[122]
		Ordered cell arrangement, improved cell sarcomere length and contraction speed	[123]
		Provides synchronized electrical/mechanical signals, more mature contractility, structure and Ca^2+^ handling	[125]

## Long-term culture *in vitro*

To obtain more mature iPSC-CMs, researchers began to explore further by increasing the *in vitro* culture time. As early as 2013, Lundy et al. [32] compared early and late iPSC-CMs and found that cells with longer *in vitro* culture times (80–120 days) were more morphologically exposed than short-term (20–40 days). The obvious differences are mainly the increase in cell size and anisotropy, the proportion of multinucleated cells increased by ten-times, and the ultrastructure showed an increase in myofibril density and a more orderly arrangement. Late iPSC-CMs contracted twice as much as early, and the rate of Ca^2+^ release and reuptake increased. Finally, electrophysiological evaluation showed that late iPSC-CMs had hyperpolarized MDP, and the AP amplitude and upstroke velocity increased. Accordingly, β-MHC and Cx43 expression is up-regulated. The present study revealed earlier that more mature iPSC-CMs phenotypes can be obtained by extending the culture time. Kamakura et al. [33] extended the *in vitro* culture time to 1 year. The iPSC-CMs myofibrils were tightly packed, forming a parallel arrangement, accompanied by the appearance of mature Z-bands, A-bands, H-bands, and I-bands. M-bands were also observed in the later stages of culture. In addition, the increase in MLC2v+/MLC2a- CMs and the decrease in MLC2v+/MLC2a+ CMs indicate the maturation of ventricular CMs.

With the development of single-cell sequencing, studies have used this technology to reveal differential gene expression and cell subpopulations during the cultivation of iPSC-CMs, and to identify candidate transcription factors that are essential for maturation. After 30 days of differentiation, multiple subgroups of iPSC-CMs rich in TBX5, NR2F2, HEY2, ISL1, JARID2, or HOPX transcription factors were identified by single-cell RNA sequencing. HOPX is a transcriptional regulator related to CM hypertrophy, and is expressed in long-term cultured iPSC-CMs. HOPX overexpression in iPSC-CMs increases cell size, while HOPX knockdown can inhibit hypertrophy and reduce the expression of multiple genes encoding sarcomeric protein maturation. In addition, the study analyzed transcriptional heterogeneity associated with changes in gene expression in the atria (NR2F2/TBX5) and ventricles (HEY2/MYL2) enrichment. Among them, NR2F2 promotes atrial-like gene expression in early differentiation, while HEY2 promotes more mature ventricular-like gene expression profiles in late differentiation. The expression levels of genes (MYL2, NAV1, SLC5A1, ACTA1) enriched in adult ventricular myocytes are overexpressed in HEY2 CMs are higher [69,70]. Through the above studies, we can have a certain understanding of the gene profile that determines cell fate and regulates more mature iPSC-CMs. In short, the advantage of long-term cultivation is that it is simple and easy to implement. However, time consumption is the main disadvantage, and some differences and potential mechanisms between adult CMs need to be further explored.

## Electrical stimulation

CMs are constantly affected by electrical signals that promote synchronous contraction in the body, and electrical activity plays an important role in the growth and development of the heart. Previous studies have pointed out that electrical stimulation can promote the differentiation of iPSC-CMs by activating the Ca/PKC/ERK pathway. Further, after 2 weeks of electrical stimulation treatment, the expression of specific myocardial structural proteins and the level of functional maturation-related genes are up-regulated. iPSC-CMs showed mature sarcomere structures, and intracellular Ca^2+^ levels increased significantly [71]. Yoshida et al. [72] gave iPSC-CMs electrical stimulation and found that cTnT expression was significantly up-regulated, CMs showed sarcomere-like structures, and its expression level was proportional to the frequency of electrical stimulation, indicating cardiac maturation. In order to better simulate the microenvironment of CMs growth, conductive silicon nanomaterials were introduced into iPSC heart spheres, combined with electrical stimulation, and by improving the endogenous electrical microenvironment, the Cx43, N-cadherin (N-Cad), and ZO-1 proteins were promoted expression and reduce the frequency of spontaneous pulsation, which is beneficial to improve the integration of grafts and host CMs. At the same time, RYR2 expression increases, which promotes the release of Ca^2+^ and regulates the contractile function of cells [73].

The above studies were based on engineered tissue and electrical stimulation. However, during development, mechanical load and electrical activity are also important determinants of CMs growth and maturity [74]. In order to explore whether the above factors can regulate the maturity of iPSC-CMs, Ruan et al. [75] found through research that pure mechanical stress, the combination of stress and electrical stimulation can increase the volume of cells and improve the arrangement of collagen fibers. The extra electrical stimulation can increase the cell’s contractility, and the cell’s ability to process Ca^2+^ is more mature. In addition, electrical stimulation can also increase the expression of GJ proteins (Cx43), N-Cad, improve the integration between them and host cells, and significantly improve mitochondrial alignment [73,76].

## Mechanical stress

The heart is essentially a mechanical pump that constantly responds to mechanical stimuli at all stages of development. Therefore, mechanical stress may be a key factor in shaping the maturity of CMs. Mechanical stimulation of iPSC-EHTs can increase the arrangement of CMs. Cyclic stress usually has a greater effect than static stress [77]. Correspondingly, the appropriate increase in EHTs contractile resistance also promotes a more mature phenotype, specifically increasing the sarcomere length, cell area and elongation, improving Ca^2+^ handling, and increasing the expression of sarcomere and ion channel-related genes [78]. In order to better induce the mature phenotype of iPSC-CMs in space and time, there have been studies that mimic the *in vivo* environment in *in vitro* based on biological information systems to regulate cell function and improve maturity. Abilez et al. [79] tested the effects of different passive stretching lengths on the structure and functional maturity of engineered heart muscle (EHM) composed of iPSC-CMs under the guidance of a computer model, and showed that when EHMs were stretched to 7 mm, the sarcomere arrangement is the most regular, the spontaneous beating slows down, the Ca^2+^ handling improves, and the expression of mature CM-related genes increases, such as β-adrenergic receptors (ADRB1), ADRB2, and calveolin-3 (CAV3), potassium inwardly-rectifying channel, subfamily J, member 2 (KCNJ2), calcium channel, voltage-dependent, L type, α 1C subunit (CACNA1C) and TNNT2.

Polydimethylsiloxane (PDMS) can provide uniaxial strain. At 8% peak-to-peak cyclic strain of 0.8 Hz, iPSC-CMs successfully attached and survived. Moreover, a more uniform sarcomere orientation perpendicular to the applied strain can be observed, which indicates that the structural maturity of the CMs is improved [80]. LaBarge et al. [76] used iPSC-CMs to prepare myocardial spheres, placed them in a device with PDMS channels, and exposed to cyclic uniaxial stretching, and performed a 7-day cyclic pull at 10% stress and 1 Hz frequency stretch. Finally, it was found that the ratio of β-MHC/α-MHC, the expression levels of MLC2v and CASQ2 were significantly increased, and the Z-shaped precursor of the Z-band was observed. The electrical stimulation alone improved cell integration, but the study did not describe the mechanical–electrical stimulation simultaneously affects the maturity of iPSC-CMs. Another study made up for this deficiency. The combined mechanical and electrical stimulation increased the expression of N-Cad and the formation of stress fibers in the iPSC-CMs membrane, but did not increase the L-type Ca^2+^ current [81]. Although mechanical–electric stimulation provides a new strategy for the maturity of CMs, the time and space control of this method needs further research to optimize, integrate, and supplement.

## Biochemical induction

Postnatal hormonal levels are considered to be a reliable source of induction of CMs maturity [42]. Thyroid hormones and glucocorticoids are essential for heart maturation during development [82,83]. Although the standard iPSC-CMs induction medium contains both factors, it has been found that adding higher concentrations of thyroid hormone or glucocorticoid to the medium can enhance the maturity of CMs [84]. After 1 week of Tri-iodo-l-thyronine (T3) culture, the size of iPSC-CMs, sarcomere length, and contractility can be significantly increased. At the same time, Ca^2+^ release and reuptake rate increase, and the expression of SERCA2a was up-regulated, and it is important that the mitochondrial maximal respiratory capacity and respiratory reserve capacity were meaningfully improved [22]. Glucocorticoid levels increase sharply in late pregnancy until the fetal organs mature. Glucocorticoid receptors (GRs) of CMs are necessary for the fetal heart structure and function to mature in the body. Glucocorticoid levels improved the contractility of primary mouse CMs, promoted the assembly of Z discs and the appearance of mature myofibrils, and increased mitochondrial activity, of which PGC-1α was considered to be a key transcription factor for the induction of glucocorticoids [83]. Based on the above studies, Parikh et al. [85] combined T3 with Dexamethasone (Dex) to intervene in iPSC-CMs, and observed that T-tube formation, Ca^2+^ release, and functional coupling between L-type Ca^2+^ channels and RYR2 were enhanced.

Although the above strategies can promote the maturity of some structures and ion channels of iPSC-CMs, the effects of energy substrates and metabolic pathways on the maturation of CMs are rarely described. Previous studies have shown that changing the medium of iPSC-CMs to a fatty acid-based medium can promote its rapid maturation into adult-like CMs, which is characterized by an increased number of mitochondria, higher oxidative metabolism, increased myofibril density, improved the Ca^2+^ handling, enhanced the contractility of CMs, increased APD50/APD90 ratio and upstroke velocity, and it is closer to the transcription characteristics of adult ventricular tissue. Sequencing results showed that fatty acid treatment up-regulated genes related to fatty acid β-oxidation and down-regulated lipid synthesis genes [86–88]. The mechanism of fatty acid induced maturation involves the inhibition of hypoxia-inducible factor 1α (HIF-1α) and its target lactate dehydrogenase A (LDHA) [45]. Previous studies have confirmed that insulin-like growth factor 1 (IGF1) regulates heart metabolism and contraction, while the combination of T3, IGF1, and DEX (TID) further improves the energy and electrophysiology of iPSC-CMs [84,89]. Based on the above studies, Gentillon et al. [90] found that the combination of TID with HIF-1α inhibitors and peroxisome proliferator activated receptor α (PPARα) agonists has a synergistic effect, further promoting fatty acid oxidation, mitochondria mature, Ca^2+^ handling, and cell contractility.

It is worth noting that the disclosure of related signaling pathways can further clarify the mature mechanism. Among the intervention strategies involved in the induction of maturation of CMs, both T3 and DEX can inhibit the mammalian target of rapamycin (mTOR) [91,92], while HIF-1α is regulated by mTOR [93], so it can be seen that mTOR plays a central role in the maturation of chemically induced CMs. mTOR is a major regulator of growth and metabolism [94], which can stimulate cell proliferation and mediate energy metabolism conversion [95]. Garbern et al. [96] used Torin1 (mTORC1/2 inhibitor) to transiently act on iPSC-CMs, which promoted the cells to express a mature phenotype, up-regulated the expression of TNNI3 and Kir2.1, and increased the maximum contractile force and oxygen consumption rate of the cells, decreased AP peak rise time and increased downstroke velocity, and the above effects were dose-dependent. Further research confirmed that the maturation of CMs is related to the cell quiescence induced by p53. The β-adrenergic receptor (β-AR) system can regulate the structure and function of CMs, and can promote the maturity of CMs to a certain extent [97]. In iPSC-CMs, with the internalization of β-AR and prolonged culture time, mature sarcomere arrangement and SR appear in the cells. However, the amount and time of β-AR stimulation need to be properly grasped, otherwise it may cause cytotoxicity [98]. In addition, based on the regulatory role of neuroregulin-1β (NRG-1β) during cardiac development, Iglesias-García et al. [99] found that NRG-1β can induce iPSC-CMs to produce AP with ventricular myocytes. Using more mature CMs for MI treatment can achieve better integration with host myocardium, inhibit myocardial remodeling, promote angiogenesis, and thereby improve cardiac function after infarction.

## Three-dimensional tissue engineering

### Co-culture

Myocardium is composed of CMs, extracellular matrix (ECM) and endothelial cells (ECs) in blood vessels. Cell growth in three-dimensional (3D) environment is more conducive to the maturation of its function and structure [100]. Among them, engineered cardiac tissues (ECTs) can induce the maturation of CMs, Masumoto et al. [101] found that the threshold of ECTs composed of iPSC-CMs, ECs, and vascular wall cells (MCs) was reduced, and it could maintain good contractility under the same intensity stimulation. It is related to more regular cell arrangement and sarcomere structure maturity in 3D cultured ECTs. Furthermore, ECTs were implanted into MI rats, and grafts and host-derived neovascularization appeared at the same time, improving cardiac function after infarction. It is worth noting that the dynamic cultivation of ECTs can provide a favorable environment for the maturity of CMs [102]. On this basis, iPSC-CMs and non-cardiac cells are dynamically cultured *in vitro* and combined with biomaterials to form a myocardial patch, which also has the effects of inducing the ultrastructure, electromechanical coupling, and Ca^2+^ handling maturation of CMs without inducing host heart arrhythmia. More importantly, the patch can also significantly improve left ventricular function, infarct area, myocardial wall stress, myocardial hypertrophy, and reduce apoptosis of myocardial cells in the surrounding area, but the integration between the graft and the host myocardium needs to be further improved [103,104]. A recent study has brought light to solve this problem. Giacomelli et al. [105] combined iPSC-CMs, cardiac fibroblasts (CFs), and ECs to construct 3D microtissues (MTs). Among them, the sarcomere structure and T-tube structure of iPSC-CMs have been improved, and energy metabolism and electrophysiology have become more mature. The mechanism that mediates maturation involves increasing the formation of GJs between iPSC-CMs and CFs and the coupling of intracellular cyclic AMP (cAMP).

Based on the secretion potential of mesenchymal stem cells (MSCs), Yoshida et al. [106] researchers observed that MSCs induced the mature phenotype of iPSC-CMs, and it was important to reveal that vascular endothelial growth factor (VEGF), basic fibroblast growth factor (bFGF), stromal cell-derived factor 1 (SDF-1) and granulocyte-macrophage colony-stimulating factor (GM-CSF) contained soluble factors are involved in this process. Although non-CMs have a positive effect on the maturation of iPSC-CMs to a certain extent, the improper ratio of non-CMs/CMs will have the opposite effect, manifested in the down-regulation of Cx43, affecting the amplitude and upstroke velocity of AP [107]. Another study showed that when CMs accounted for 50% of the total number of cells, ECTs showed synchronous spontaneous beating, accompanied by an increase in conduction velocity with the increase in the proportion of CMs. However, ECTs composed of 90% CMs cannot form a stable structure. ECTs containing 25 or 50% CMs mainly expressed collagen and fibronectin, while ECTs containing 70% CMs mainly expressed laminin and exhibited the highest contractile/diastolic characteristics [108].

### ECM

ECM is an important regulator of homeostasis at the level of cells, tissues, and organs. The dynamics of actin structure in different cell types are closely related to the characteristics and stiffness of ECM. In the heart, the composition and distribution of ECM follow the process of cardiac development changes [109], and affect cell contraction [110] and mitochondrial function [111]. The maturation of PSC-CMs can be achieved by adding ECM proteins and adjusting substrate stiffness [112]. One study pointed out that iPSC-CMs plated in a single layer on ECM based on PDMS + Matrigel have faster impulse propagation velocities and have mature CM AP profiles, including hyperpolarized diastolic potential and rapid AP upstroke velocity. In addition, the optimal ECM promoted hypertrophic growth of CMs and the expression of key mature sarcolemmal (SCN5A, Kir2.1, and Cx43) and myofilament markers (cTnI). The maturation process relies on activation of integrin signaling pathways: neutralization of β1 integrin receptors via blocking antibodies and pharmacological blockade of focal adhesion kinase activation prevented structural maturation [113]. Researchers demonstrated that 3D cardiac ECM promote increased expression of calcium-handling genes, Junctin, CaV1.2, NCX1, HCN4, SERCA2a, Triadin, and CASQ2. In addition, 3D adult cardiac ECM can increase the calcium signal (amplitude) and dynamics (maximum upstroke and downstroke) of iPSC-CMs more than 2D. Cells in 3D culture were also more responsive to caffeine, likely reflecting an increased availability of calcium in the SR [114]. The presence of ECM can also induce the maturation of CMs structure [115].

At present, there are researches that use decellularized cardiac ECM (dcECM) from many different animal models to promote the maturation of CMs. The advantages of dcECMs include the anisotropic arrangement of natural heart, essential components of cardiac ECM and development related vasculature [116]. Schwan et al. [117] installed dcECMs on a special fixture system and then inoculated them with iPSC-CMs and found that they can improve the contractile strength and sarcoid tissue of CMs, and enhance the Cx43, N-Cad, and cardiac troponin expression. Another study created a more clinically meaningful model in which iPSC-CMs were inoculated into an adult decellularized heart and placed in a bioreactor system that provided perfusion through the coronary arteries and left ventricular wall stress using a balloon for 14 days, CMs were observed to contract easily, have metabolic activity, and express related myocardial markers, but there was a problem of uneven cell maturity [118]. As mentioned earlier, some researchers compared the induction of iPSC-CMs maturation by decellularized embryos and adult myocardium, and the results showed that key mature genes have higher expression levels in adult dcECMs [114]. Reasons include that because the mouse heart stopped cardiogenesis on the 18th day of the embryo, dcECMs in late-developing embryos may no longer provide ideal development signals related to iPSC-CMs proliferation and maturity [42,119], and ECM is in a dynamic change during cardiac generation. Therefore, the effect of embryonic dcECMs on iPSC-CMs maturation is weaker than that of adult dcECMs.

### Biomaterials

ECM-derived hydrogels have been widely studied as cardiac tissue substitutes. However, they show premature degradation, and their stiffness is usually orders of magnitude lower than that of natural heart tissue. As mentioned earlier, the stiffness of a suitable substrate is critical for the maturation of iPSC-CMs. Studies have wrapped iPSC-CMs in chemically crosslinked gelatin hydrogels (1.25g × 10mL) with adjustable stiffness and degradability. To investigate the effect of hydrogel properties on the maturity of CMs, compared with high stiffness (16kPa)/slow degradation hydrogels, it was found that the formation of intercellular networks, the expression of α-actin and Cx43, and the contraction speed were increased in low stiffness (2kPa)/fast degradation and medium stiffness (9kPa)/moderate degradation hydrogels. Only the cells in the 9 kPa hydrogel showed organized sarcomere structure and significantly increased contractility [120]. This again demonstrates that mimicking the microenvironment of CMs and increasing intercellular connectivity can help improve iPSC-CMs maturation. In order to reduce the implantation of non-conductive matrix and increase the incidence of arrhythmia, Roshanbinfar et al. [121] developed a conductive bio-hybrid hydrogel. Studies have confirmed that iPSC-CMs show better contraction amplitude and enhanced Cx43 expression on conductive hydrogels compared to cells cultured on a common substrate. The sarcomere tissue improved and the length increased from 1.3μm to 1.7μm, suggesting the maturation of iPSC-CMs. In addition, 3D ECTs exhibit enhanced Ca^2+^ handling capabilities and positive responses to external electric and drug stimulation, providing a good biomaterial for stem cell-based cardiac tissue engineering.

Recently, research has also developed a new type of biomaterial that integrates differentiation and maturity induction, and compares the effects of binary colloidal crystals (BCCs) composed of different components on iPSC-CMs. 3D spheres were formed in BCC (5PM) composed of silicon oxide particles and 0.4μm polymethyl methacrylate, and 5PM improved the maturity of cell structure, electrophysiology and other aspects, and promoted the transition from E-Cad to N-Cad. Perhaps a potential mechanism for 5PM to induce maturation [122]. However, the organized arrangement of CMs is the key to maintaining the mechanical properties of the heart. In order to better simulate the highly ordered physiological arrangement and function of natural CMs, so that CMs can be assembled in a controlled manner, Wanjare et al. [123] demonstrated that aligned microfibrous scaffolds induced iPSC-CMs alignment along the direction of the aligned microfibers after CMs seeding, as well as promoted greater CMs maturation by increasing the sarcomeric length and gene expression of MYH7, in comparison to randomly oriented scaffolds. Furthermore, the benefit of scaffold anisotropy was evident in the significantly higher maximum contraction velocity of iCMs on the aligned scaffolds, compared to randomly oriented scaffolds. Similarly, a bionic microchip with an onion-like epithelial structure can provide a framework for cell growth, and also has the ability to induce the maturation of iPSC-CMs structure and improve Ca^2+^ handling capacity [124]. In addition, in order to further induce the synchronized contractions of CMs, another study incorporated electrically conductive silicon nanowires (e-SiNWs) into iPSC-CMs spheres to promote the formation of conductive networks and provide synchronization and improvement electrical/mechanical signals to promote the contractile maturation of CMs, including a 55% increase in contraction amplitude, an increase in the overall calcium level amplitude (F/F0), and a faster time to reach the peak of calcium transients, Cx43, α-sarcomeric actinin (α-SA) and cTnI expression were significantly up-regulated, and Ca^2+^ handling channels were significantly improved. Further e-SiNWs-enhanced cell spheroids were cultured in a 3D microenvironment, and the sarcomere length and Z-band width were significantly improved, with Z-bands similar to adult CMs [125].

## Conclusion

At present, iPSC-CMs have shown broad application prospects in many fields, such as pharmacological research, heart disease models, and treatment of MI. However, in order to realize this potential, we need to conduct corresponding research to promote their maturity. The maturation of CMs *in vivo* is regulated by a variety of factors, including electrical signals, mechanical signals, adhesion, biochemistry, and cell-to-cell interactions. It is natural to want to break down these influencing factors, decipher the signaling pathways that control phenotypes, and try to use them to induce this process [27]. Although there are many intervention strategies to push iPSC-CMs to a more mature phenotype, maturation is a complex feature. Therefore, in order to obtain a better maturation status of iPSC-CMs, it may be necessary to expose the cells to multiple regulatory means simultaneously [126]. As mentioned earlier, a number of different intervention strategies can induce more mature phenotypes. However, an appropriate approach may require not only a combination approach, but also the timing, sequence, intensity, and duration of interventions to mediate and reflect the maturation process. For example, the frequency and duration of EHT combined with electrical stimulation, stress when combined with mechanical stimulation, different cell ratios during co-culture, matrix hardness, source, and shape. With the current deepening of bioengineering research, intervention methods based on EHT and biomaterials not only simulate the microenvironment *in vivo* for cell growth, but also provide positive regulation for the maturation of CMs, so they can be used as new strategies of induction *in vitro*.

Nevertheless, there are still some issues to be resolved. On the one hand, for cell transplantation, adult CMs cannot be transplanted into damaged myocardium because they cannot survive to form new myocardium, but fetal and neonatal cells are more suitable [127], so this may be disadvantageous for regenerative medicine applications. Therefore, the maturation of the heart may be a double-edged sword. For the purpose of cell transplantation, the best choice may be to obtain the phenotype of CMs with the best mature state that has not yet been determined, for the purpose of optimal transplantation and functional improvement. Secondly, it is the scalability of mature iPSC-CMs production. Although there are many effective methods to improve maturity, due to the fact that each patient needs about billions of cells, and the complexity of the culture conditions is inversely proportional to scalability, cost, and reproducibility. The number of CMs required for regenerative medicine cannot be generated in a high-throughput manner. Therefore, it is expected that new methods can be introduced to complement the existing ones, thereby achieving higher application value in inducing the maturation of iPSC-CMs and cell transplantation. For example, the combination of ascorbic acid and growth factors can effectively improve the differentiation efficiency and maturity of iPSC-CMs, telomere length can have an impact on cell fate determination, and improve differentiation yield [128,129].

In conclusion, the technology that induces the maturity of iPSC-CMs opens a new chapter for subsequent practical applications. Although there are still some limitations, it is believed that with further research and expansion, large-scale mature iPSC-CMs can be developed for application in regenerative medicine.

## Abbreviations

ADSC: adipose-derived stem cell
AP: action potential
CF: cardiac fibroblast
CM: cardiomyocyte
cTnT: cardiac troponin T
CVD: cardiovascular disease
Cx43: connexin 43
dcECM: decellularized cardiac ECM
Dex: Dexamethasone
EC: endothelial cell
ECM: extracellular matrix
ECT: engineered cardiac tissue
EHM: engineered heart muscle
ESC: embryonic stem cell
e-SiNW: electrically conductive silicon nanowire
GJ: gap junction
HIF-1α: hypoxia-inducible factor 1α
IGF1: insulin-like growth factor 1
iPSC: induced pluripotent stem cell
LTCC: L-type calcium channel
MDP: maximum diastolic potential
MLC: myosin light chain
MP: membrane potential
MSC: mesenchymal stem cell
mTOR: mammalian target of rapamycin
MYH7: myosin heavy chain 7
NRG-1β: neuroregulin-1β
N-Cad: N-cadherin
PDMS: polydimethylsiloxane
RMP: resting MP
RYR: ryanodine receptor
SC: stem cell
SR: sarcoplasmic reticulum
TID: combination of T3, IGF1, and DEX
T3: tri-iodo-l-thyronine
β-AR: β-adrenergic receptor
β-MHC: β-myosin heavy chain
3D: three-dimensional
